# Comprehensive analysis reveals the prognostic and immunogenic characteristics of DNA methylation regulators in lung adenocarcinoma

**DOI:** 10.1186/s12931-024-02695-4

**Published:** 2024-02-05

**Authors:** Jing Huang, Chujian Huang, Can Huang, Zichang Xiang, Yao Ni, Jian Zeng, Songhua Cai

**Affiliations:** 1 Department of Thoracic Surgery, Sun Yat-sen Memorial Hospital, Sun Yat-sen University, 107 Yanjiang West Road, Guangzhou, 510120 China; 2 Department of Thoracic Surgery, National Cancer Center/National Clinical Research Center for Cancer/Cancer Hospital & Shenzhen Hospital, Chinese Academy of Medical Sciences and Peking Union Medical College, Shenzhen, 518116 China; 3 Eight-year MD program, Peking Union Medical College, Chinese Academy of Medical Sciences, Peking Union Medical College, Beijing, 100087 China; 4 Shenzhen University Medical School, Shenzhen, 518055 Guangdong China; 5 Department of Anesthesiology, Longgang District Central Hospital of Shenzhen, Shenzhen, 518116 Guangdong China

**Keywords:** Lung adenocarcinoma, DNA methylation regulator, Tumor microenvironment, Prognosis, Immune response

## Abstract

DNA methylation regulators (DMRs) play a key role in DNA methylation, thus mediating tumor occurrence, metastasis, and immunomodulation. However, the effects of DMRs on clinical outcomes and immunotherapy response remain unexplored in lung adenocarcinoma (LUAD). In this study, eight LUAD cohorts and one immunotherapeutic cohort of lung cancer were utilized. We constructed a DNA methylation regulators-related signature (DMRRS) using univariate and multivariate COX regression analysis. The DMRRS-defined low-risk group was preferentially associated with favorable prognosis, tumor-inhibiting microenvironment, more sensitivity to several targeted therapy drugs, and better immune response. Afterward, the prognostic value and predictive potential in immunotherapy response were validated. Collectively, our findings uncovered that the DMRRS was closely associated with the tumor immune microenvironment and could effectively predict the clinical outcome and immune response of LUAD patients.

**Supplementary Information:**

The online version contains supplementary material available at 10.1186/s12931-024-02695-4.

## Introduction

Lung cancer remains one of the leading causes of cancer-related deaths worldwide [31], among which more than 40% of patients have lung adenocarcinoma (LUAD) [30]. Numerous therapies, such as surgery, radiotherapy, chemotherapy, molecular targeted therapy, and immunotherapy, have been developed for the clinical treatment of LUAD in recent decades. However, the 5-year survival rate of advanced LUAD is still less than 25% due to the complexity of the tumor formation mechanism [8, 32]. Therefore, it is urgent to find new prognostic biomarkers or therapeutic targets.

Immune checkpoint blocker therapy has achieved impressive success in treating various tumor types [1, 7, 16]. However, only a few patients can benefit from immunotherapy [35]. To identify patients who respond well to immunotherapy, the researchers have made great efforts in exploring potential biomarkers. The tumor mutation burden (TMB), microsatellite instability (MSI) status, PD-L1 expression, and some mutated genes have shown a great future [14, 25, 27]. Unfortunately, the predictive power of these markers varies due to tumor heterogeneity. Better biomarkers may be uncovered by focusing on specific cancer types.

As one of the most important epigenetic modifications, DNA methylation plays a crucial role in various biological processes [29]. The potential prognosis of DNA methylation regulators has been noted in several types of cancer [41, 43]. Nevertheless, the value of DNA methylation regulators in LUAD remains largely unknown. In addition, there is growing evidence of a link between DNA methylation and tumor immunity [24, 33]. For example, TET1 mutation has been strongly associated with increased tumor immunogenicity and could be used as a potential biomarker for immune checkpoint blocker therapy in multiple cancers [38]. A valuable methylation score has been established based on DNA methylation regulators, which has helped develop personalized immunotherapy strategies for gastric cancer [23].

This study aimed to conduct a comprehensive analysis of DNA methylation regulators in LUAD based on the TCGA and GEO databases. Toward this goal, a DNA methylation regulators-related signature (DMRRS) was constructed to predict the prognosis of patients with LUAD effectively. Then the relationships between immune scores, immune cell infiltration, chemotherapy and targeted therapy sensitivity, immune response, and risk signature were thoroughly analyzed.

## Materials and methods

### Dataset source and preprocessing

Seven microarray datasets (GSE19188, GSE30219, GSE31210, GSE3141, GSE37745, GSE50081, and GSE72094) were obtained from Gene Expression Omnibus (GEO) database. The expression matrices of the above datasets and corresponding clinical data were downloaded using the GEOquery R package [5]. Since GSE19188, GSE30219, GSE31210, GSE37745, and GSE50081 were all conducted in the same platform: Affymetrix Human Genome U133 Plus 2.0 Array, we then processed the raw CEL data of these five datasets by the robust multichip average (RMA) algorithm for background correction and normalization [10]. Finally, using the ComBat function of the sva R package for batch removal, we integrated them into a large GEO cohort and referred to it as the Large-GEO cohort hereinafter [19].

The RNA sequencing data (FPKM value) of gene expression in TCGA databases were downloaded from the Genomic Data Commons (GDC, https://portal.gdc.cancer.gov/) using the R package TCGAbiolinks [3]. The corresponding clinical information of LUAD was downloaded from Xena public data hubs. The data of 533 LUAD samples and 59 adjacent normal tissues were downloaded on April 10, 2021. The following inclusion criteria were used: (1) histologically confirmed LUAD; (2) simultaneously available information on mRNA expression profile data and OS and (3) Survival time ≥ 30 days. Lastly, 490 patients with LUAD and the corresponding clinicopathological information, including age, gender, TNM staging, and grade, were enrolled for further analysis.

Publicly available immunotherapeutic datasets of lung cancer with both gene expression and corresponding clinical data were thoroughly searched. We just found the GSE126044 cohort, containing 16 lung cancer patients treated with anti-PD-1 antibody.

The detailed clinical information of the above datasets is shown in **Supplementary Table **1.

### Multi-omic landscape of DNA methylation regulators in TCGA-LUAD

About 20 DNA methylation regulators were collected from previously published studies, including three writers (DNMT1, DNMT3A, DNMT3B), three erasers (TET1, TET2, TET3), and 14 readers (MBD1, MBD2, MBD3, MBD4, ZBTB33, ZBTB38, ZBTB4, UHRF1, UHRF2, MECP2, UNG, TDG, NTHL1, SMUG1). The somatic mutation and Copy Number Variation (CNV) data were acquired from the TCGA database and further analyzed. In short, we downloaded the mutation and CNV data using the GDCquery package. The mutation data was visualized by the maftools package. As for CNV data, we thresholded them by a noise cutoff of 0.2. Genes with CNV values smaller than − 0.2 were categorized as “loss”, while larger than 0.2 were as “gain”. Next, the differential expression of those regulators was compared between the tumor tissues and adjacent normal pairs.

### Construction of the DNA methylation regulators-related signature (DMRRS)

We first performed univariate Cox regression analysis in both TCGA and Large-GEO cohorts to evaluate the DNA methylation regulators’ prognostic value. Then, we utilized multivariate Cox regression analysis to construct the powerful prognostic signature in the Large GEO training cohort with the backward stepwise regression method. A risk score for each patient was calculated as the sum of each gene’s score, which was generated using the following formula:

$${Risk} {score} = \sum _{i=1}^{n}Coef\left(i\right)*Expression\left(i\right)$$

Where n represents the number of genes, *Coef(i)* is the coefficient of relative prognostic genes in the model, and *Expression(i)* is the expression of each selected gene. The sensitivity and specificity of the prognostic signature were further accessed by receiver operating characteristic (ROC) curves and the area under the ROC curves (AUC values). According to this equation, the risk score of each patient was calculated in the Large-GEO training, TCGA, GSE3141, and GSE72094 cohorts. The patients were then divided into high- and low-risk groups using the surv-cutpoint function in the survminer package.

### Nomogram development and evaluation

We utilized the univariate and multivariate Cox regression analysis to choose the independent prognostic factors in TCGA-LUAD. Based on the TNM grade and risk score, we developed the nomogram using the rms R package. To evaluate the prediction accuracy of the nomogram, we calculated Harrell’s consistency index and plotted the calibration curves.

### Gene set variation analysis (GSVA) and functional annotation

To investigate the biological process of two risk groups, Gene set variation analysis (GSVA) was performed using the GSVA R package [13]. Differential analysis was then conducted to determine the significantly enriched pathways in each group. The gene sets of ‘c2.cp.kegg.v7.4.symbols’ were downloaded from the MSigDB database for running GSVA analysis. Adjusted P with a value less than 0.05 was considered statistically significant.

### Estimation of immune score and immune cell infiltrates

We used the ESTIMATE algorithm to calculate each patient’s stromal and immune scores [40]. The fraction of 22 immune cell types for each sample was yielded by estimating relative subsets of RNA transcripts (CIBERSORT; https://cibersort.stanford.edu/). The algorithm of 1,000 permutations was adopted. Only samples with a CIBERSORT p of < 0.05 were included to perform the subsequent analysis. Correlation between different risk groups and abundances of each cell type were analyzed and visualized by radar chart.

### Tumor immune estimation resource (TIMER) database

The TIMER database was used to evaluate the correlation between DMRRS-related genes and immune cell infiltrates in the tumor microenvironment. In addition, using the Mutation module of the TIMER database, we compared the contents of immune cells in wild and mutant types of DMRRS-related genes.

### Tumor immune single cell hub database

The Tumor Immune Single-Cell Hub (TISCH) (http://tisch.comp-genomics.org) provided a detailed characterization of the immune system heterogeneity in tumors at the single-cell level [34]. Our study used the TISCH database to evaluate the expression of DMRRS-related genes in different immune cells.

### Estimation of drug sensitivity

The pRRophetic R package was used to estimate drug sensitivity, determined by the IC50 of compounds [11].

### Assessment of immunophenoscore (IPS)

We downloaded the immunophenoscore (IPS) from The Cancer Immunome Atlas (TCIA) to validate the predicting roles of DMRRS in the immune checkpoint inhibitor (ICI) treatment. A higher IPS score indicates a better immunotherapy response [2].

### Clinical specimens and immunohistochemistry (IHC)

We retrospectively collected 18 paraffin-embedded LUAD specimens and 18 adjacent normal tissues from the National Cancer Center/National Clinical Research Center for Cancer/Cancer Hospital & Shenzhen Hospital (Shenzhen, China) to compare the differential expression of UHRF1 and MECP2. For survival analysis, we further enrolled 100 tumors for IHC and collected clinical information including age, gender, TNM stage, smoking or not, and disease-free survival (DFS). The cutoff value was generated using the surv-cutpoint R function. Informed consent was obtained from all patients. This study was approved by the Ethics and Research Committees of the National Cancer Center/Cancer Hospital & Shenzhen Hospital, the Chinese Academy of Medical Sciences, and Peking Union Medical College.

After deparaffinization, rehydration and antigen retrieval, the samples were incubated with primary antibodies against MECP2 (Proteintech, 21402-1-AP, 1: 100) and UHRF1 (Proteintech, 10861-1-AP, 1: 100) at 4 °C overnight. The slides were then incubated with anti-rabbit secondary antibody and followed by chromogen DAB staining and haematoxylin counterstaining. The expression of selected markers was scored by percent of positive cells (1 − 100%) and staining intensity (1 = weak, 2 = moderate, 3 = strong). A final histoscore (H-score) was derived as: H-score = (percentage of weak intensity ×1) + (percentage of moderate intensity × 2) + (percentage of strong intensity ×3), yielding a range of possible H-scores of 0 to 300 [6]. Two pathologists who were blind to the information of patients independently validated the IHC results.

### Statistical analysis

All statistical analyses were performed using SPSS 26.0 (Chicago, IL, USA) and R software version 4.0.5 (http://www.r-project.org). Continuous parameters were expressed as mean value ± standard deviation (SD) and were compared by the Mann-Whitney U test and Kruskal–Wallis test between different groups. For categorical parameters, proportions were compared by Pearson’s Chi-square test. Pearson’s correlations were employed to assess associations between two variables. Survival curves were plotted by the Kaplan-Meier method and were compared by log-rank test. A *P* value less than 0.05 was considered statistically significant.

## Results

### Multi-omic characteristics of DNA methylation regulators in TCGA-LUAD

**Fig. 1:**
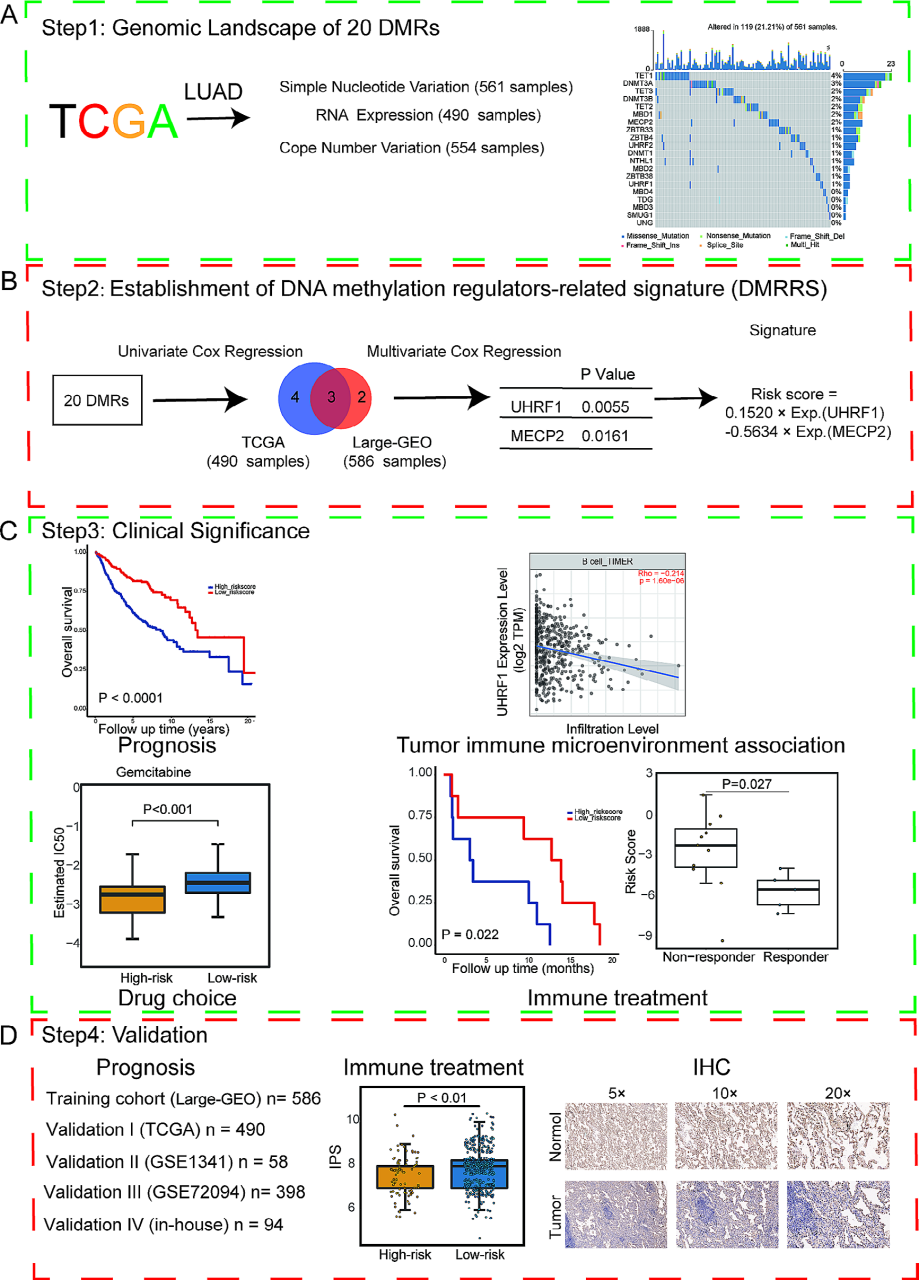
Study Design and Workflow of The Study. (**A**) Step 1: Genomic landscape of 20 DMRs, including simple nucleotide variation, copy number variation and RNA expression analysis. (**B**) Step 2: Establishment of DMRRS. (**C**) Step 3: Clinical significance of DMRRS. The analysis of prognostic value, tumor microenvironment association, chemotherapy and targeted therapy drug sensitivity, and immune response in DMRRS-defined subgroups. (**D**) Step 4: Validation of prognostic value and predicting potential of immune response of DMRRS. IHC validation of the abnormal expression of DMRRS’ two genes between normal and tumor tissues. DMRs: DNA methylation regulators. DMRRS: DNA methylation regulators-related signature

**Fig. 2:**
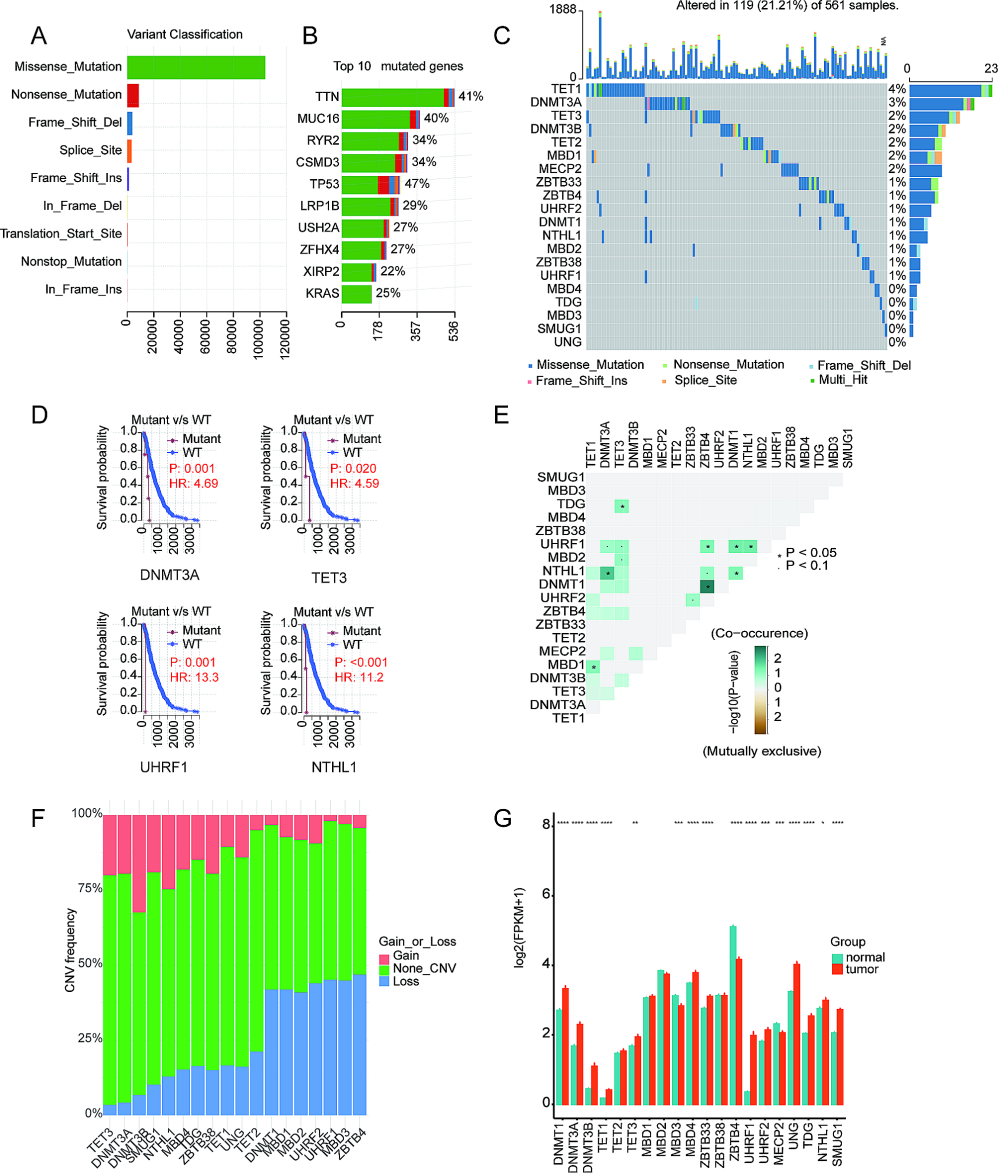
Multi-Omic Landscape of DNA Methylation Regulators in TCGA-LUAD Cohort. (**A**) Classification of mutations of different DNA methylation regulators genes. (**B**) the top 10 mutated genes. (**C**) Mutations of the 20 DNA regulators genes. (**D**) The survival curve of mutated regulators genes. (**E**) The mutation co-occurrence features of DNA methylation regulators. (**F**) The CNV features of DNA methylation regulators. (**G**) Expression levels of 20 DNA methylation regulators in 57 tumor and adjacent normal pairs. ∗*p* < 0.05, ∗∗*p* < 0.01, ∗∗∗*p* < 0.001 and ∗∗∗∗*p* < 0.0001. HR: hazard ratio

The study flowchart is depicted in Fig. 1. In the TCGA-LUAD cohort, several important oncogenes like TP53 and KRAS were among the top mutated genes, with missense mutations being the most frequent mutation event (Fig. 2A and B). Despite the lower mutation rates, the mutant of DNMT3A, TET3, UHRF1, and NTHL1 showed worse overall survival (OS) (Fig. 2C and D). Several co-occurrence relationships among the DNA methylation regulators were revealed, such as DNMT3A and NTHL1, DNMT1 and ZBTB4, and so on (Fig. 2E). As for CNV, readers like UHRF1-2, MBD1-3, and ZBTB4 displayed the highest frequency of copy number losses, while writes and erasers tend to have prevalent copy number gains (Fig. 2F).

To investigate the expression features, we systematically analyzed 57 LUAD tissues and adjacent normal tissues from TCGA. The results showed that most DNA regulators were differently expressed between LUAD and normal tissues (Fig. 2G). The expression levels of three writers (DNMT1, DNMT3A, DNMT3B), two erasers (TET1, TET3), and eight readers (MBD4, ZBTB33, UHRF1, UHRF2, UNG, TDG, NTHL1, SMUG1) were dramatically higher in tumor tissues (*p* < 0.05). While MBD3, ZBTB4, and MECP2 were markedly lower in LUAD tissues than in normal tissues (*p* < 0.05). No statistically significant difference was evident regarding the expression level of TET2, MBD1, MBD2, and ZBTB38.

### Construction of DNA methylation regulators-related signature (DMRRS)

**Fig. 3:**
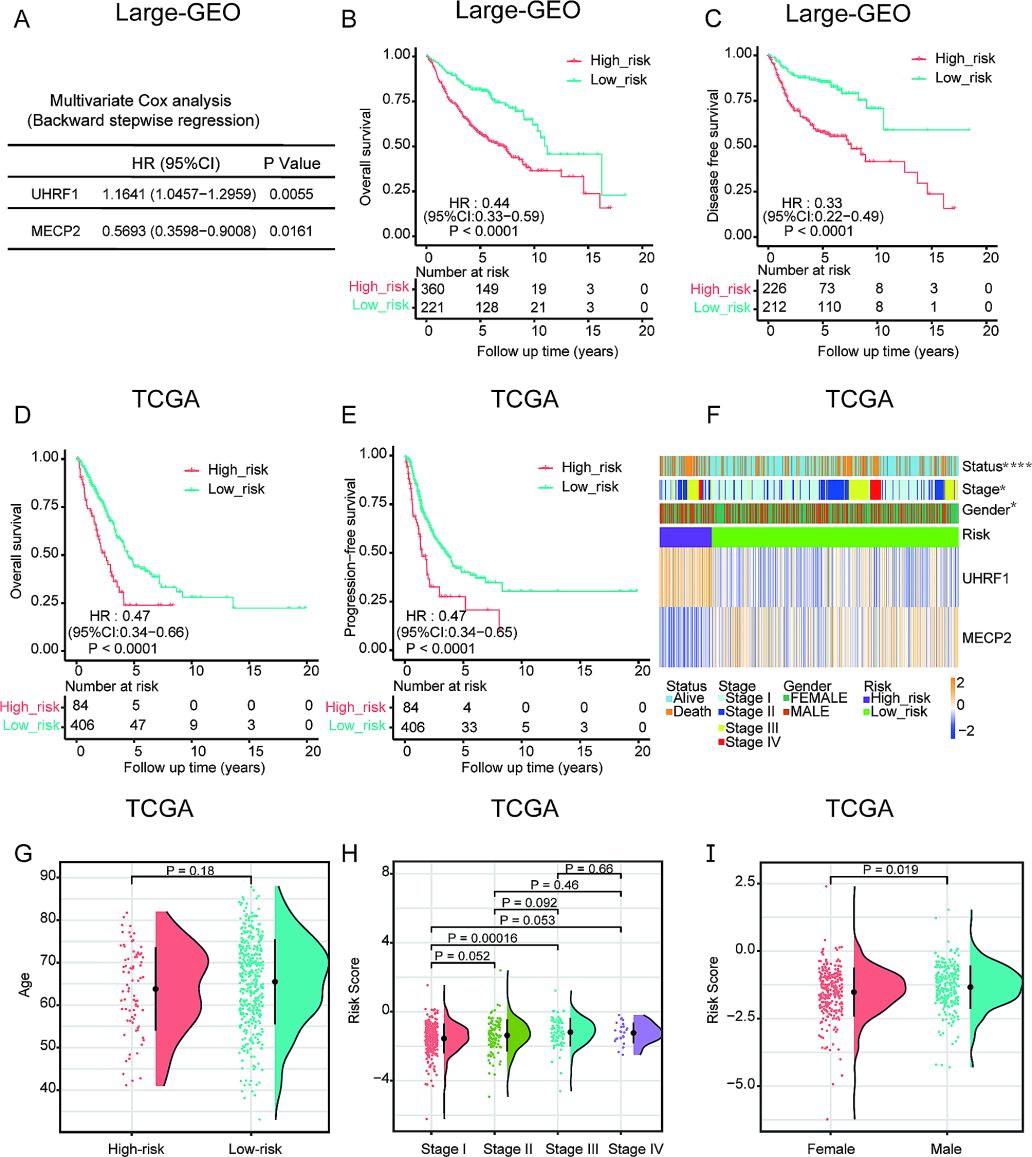
Construction of DMRRS in Large-GEO and Clinical Features of DMMRS-Defined Subgroups in TCGA Cohort. (**A**) Using multivariate Cox regression, two DNA methylation regulators were selected for risk coefficient calculation. (**B**) Kaplan-Meier curves of OS for patients with LUAD based on the risk score in the Large-GEO cohort. (**C**) Kaplan-Meier curves of DFS for patients with LUAD based on the risk score in the Large-GEO cohort. (**D**) Kaplan-Meier curves of OS for patients with LUAD based on the risk score in the TCGA cohort. (**E**) Kaplan-Meier curves of PFS for patients with LUAD based on the risk score in the TCGA cohort. (**F**) Heatmap and clinical features of high- and low-risk groups in TCGA cohort. (**G-I**) Relationship between risk scores and (**G**) Age, (**H**) Stage, and (**I**) Gender in TCGA cohort.OS: overall survival; DFS: disease-free survival; PFS: progression-free survival. ∗*p* < 0.05, ∗∗*p* < 0.01, ∗∗∗*p* < 0.001 and ∗∗∗∗*p* < 0.0001

Five regulators in Large-GEO data (MECP2, ZBTB4, NTHL1, UHRF1, and MBD4) and seven regulators (TET2, MECP2, ZBTB4, UNG, SMUG1, TDG, and UHRF1) in TCGA were significantly related to the OS after univariate Cox regression analysis (**Supplementary Tables **2 and 3). Three genes were linked to patients’ survival in both cohorts. So, we next used the three intersected genes to build the prognostic signature by the multivariate Cox regression analysis in the Large-GEO cohort with the backward stepwise method **(**Fig. 3A**)**. Only two regulators, UHRF1 and MECP2, were included in the DMRRS, and the equation was as follows: (Risk score = 0.1520 × UHRF1 expression level) − (0.5634 × MECP2 expression level). Afterward, all LUAD patients were divided into low-risk and high-risk groups based on the optimal cutoff value of the risk score. Kaplan–Meier survival analysis revealed that the OS of the high-risk group was lower than that of the low-risk group in the Large-GEO cohort (Fig. 3B**)**. Besides, in the Large-GEO dataset, the disease-free survival (DFS) of the low-risk group was also significantly higher than the other group **(**Fig. 3C**)**. The Receiver operating characteristic (ROC) curve was used to assess the prognostic accuracy of identified risk signatures, with the 1-, 3- and 5-year AUC values for the two risk signatures being 0.620, 0.622, and 0.625, respectively **(Supplementary Fig. 1A)**.

### Clinical characteristics associated with DMRRS

The clinical characteristics of patients in different risk groups were further evaluated in the TCGA cohort. The low-risk patients showed better clinical outcomes as expected in the cohort (Fig. 3D and E).

The heatmap demonstrated the expression levels of two DNA methylation regulators between high- and low-risk groups in TCGA-LUAD (Fig. 3F). The expression levels of UHRF1 were typically higher in the high-risk group than those of the low-risk group, while MECP2 expression was lower in the high-risk group. The difference in gender and TNM stages between the two groups was significant. As shown in Fig. 3G-I, male patients had an increased risk score level than female patients. Patients in stage I tended to have a lower score when compared with stage II-IV. We then performed OS analysis between high- and low-risk subgroups regarding different clinical features, including age, gender, and stages. The low-risk group had obviously better OS than the high-risk group whatever the age or the gender (**Supplementary Fig. **1B and 1 C). As for the stage, Patients with stage I tend to have more influence on the OS than the lower scores. However, the trend of better OS in the low-risk group still holds (**Supplementary Fig. **1D).

### Construction and evaluation of the prognostic nomogram

**Fig. 4:**
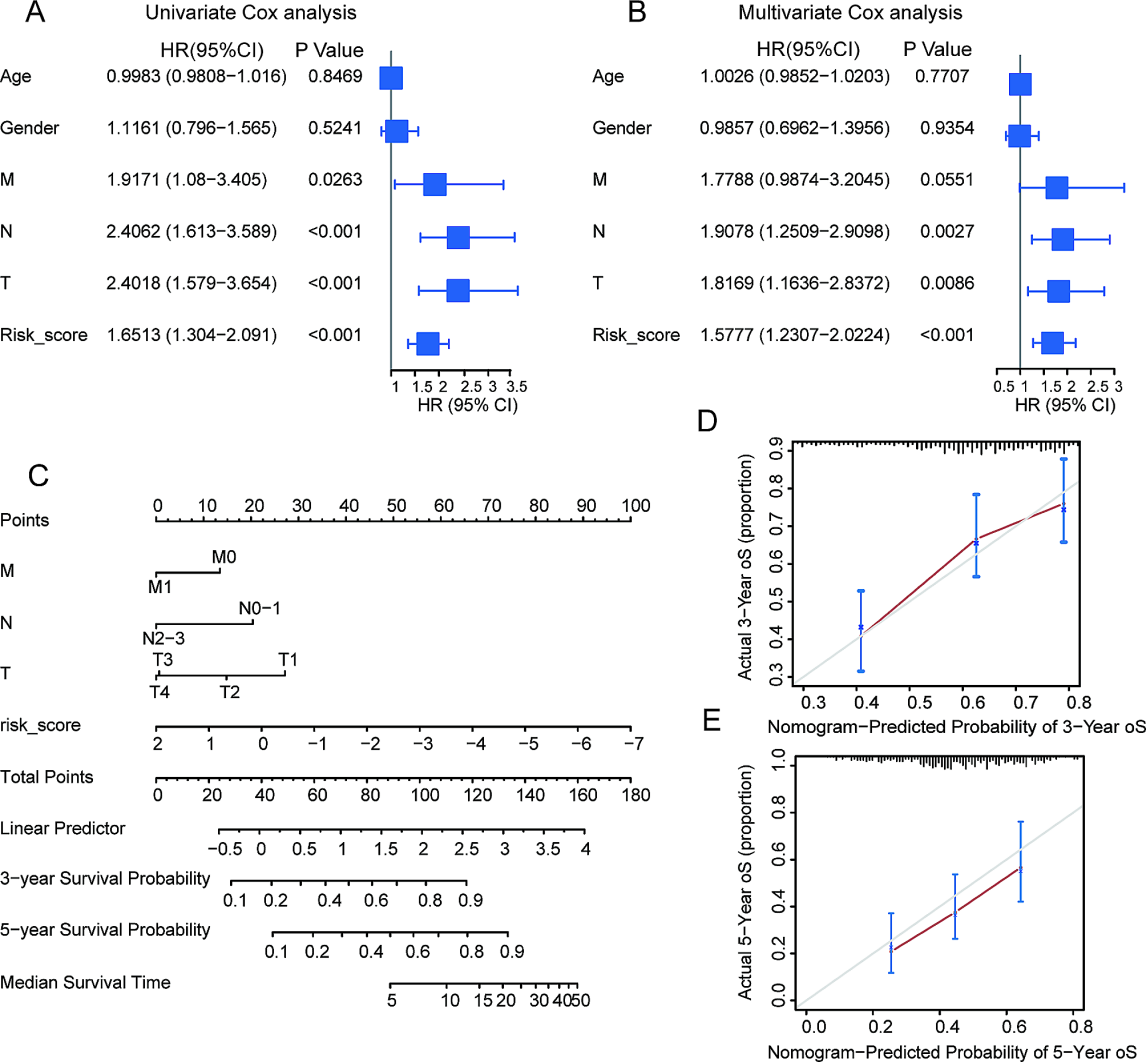
Construction and Validation of Nomogram. (**A**) Forest plot of univariate Cox analysis results. (**B**) Forest plot of multivariate Cox analysis results. (**C**) Nomogram for predicting 3- and 5-year overall survival of OC patients. (**D-E**) Calibration curves of the nomogram prediction of 3 years (**D**) and 5 years (**E**) OS of patients

To illustrate the prognostic benefits of the DMRRS-defined low-risk patients weren’t just because of lower tumor stages, we performed the univariate and multivariate Cox regression analyses in the TCGA dataset. The results demonstrated that DMRRS was an independent prognostic factor (Fig. 4A and B). Thus, we fabricated a nomogram based on clinical risk characteristics, including TNM stages and DMRRS-defined risk scores, to predict 3-and 5-year OS probability. The risk model showed predominant predictive ability (Fig. 4C). The C index of the nomogram model was 0.69, and correlation charts displayed ideal consistency (Fig. 4D and E). Collectively, those results above indicated a promising prognostic significance of DMRRS.

### Correlation between the tumor microenvironment and DMRRS

**Fig. 5:**
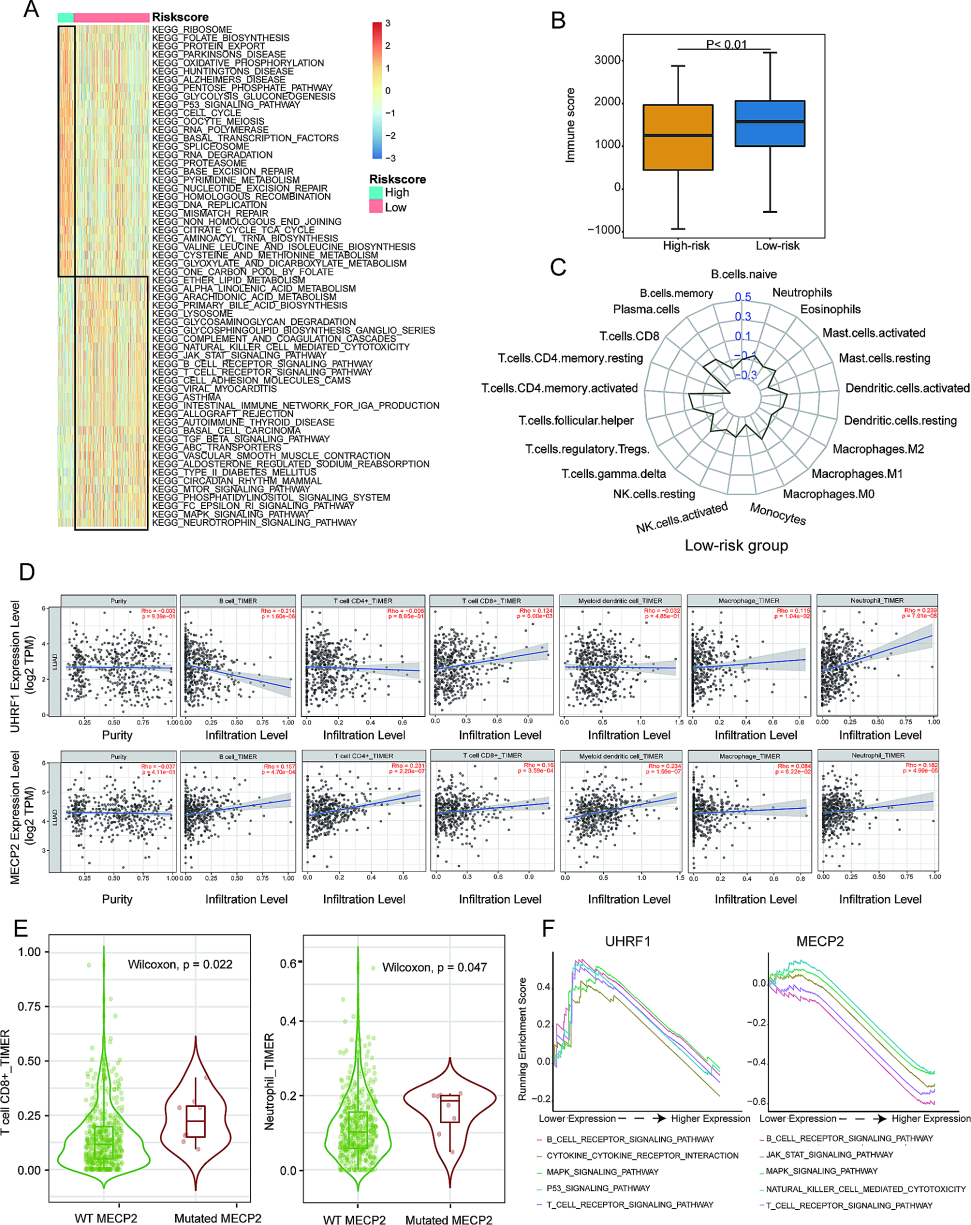
Correlation between DMRRS and Tumor Microenvironment. (**A**) GSVA results to distinguish the pathways between high- and low-risk groups. (**B**) Immune scores difference between two risk groups. (**C**) Correlation between low-risk groups and 22 immune cells. (**D**) Correlation analysis of UHRF1 and MECP2 with the infiltration level of the six main immune cells after adjusting for the purity. (**E**) The significant difference in CD8 + T cells and neutrophil infiltration levels between mutant and wild type. (**F**) The enrichment pathways of GSEA varied with different expression of UHRF1 or MECP2

To reveal the different biological behaviors of risk clusters in LUAD, we then compared GSVA scores in high- and low-risk groups using the limma package [28]. Intriguingly, many cancer-related pathways like cell cycle, DNA replication, and p53 signaling pathways were excessively activated in the high-risk group. In contrast, several immune-related pathways were activated in the low-risk group, such as the B/T cell receptor signaling pathway, natural killer cell-mediated cytotoxicity, and complement and coagulation cascades (Fig. 5A).

Next, we analyzed the relationship between immune scores, infiltration levels of immune cell types, and DMRRS-defined subgroups. The results showed that the high-risk patients had remarkably decreased immune scores (Fig. 5B). In addition, patients in the high-risk group were closely related to the macrophage M1 cell and activated Mast cells (**Supplementary Fig. 1E**), while low-risk patients tended to associate with adaptive immune cells such as activated CD4 T cells and CD8 T cells (Fig. 5C). It was rational to speculate that the DMRRS-defined low-risk group might be highly associated with the active tumor immune microenvironment.

We then analyzed the correlation of the two components of DMRRS: UHRF1 and MECP2, with immune cell infiltration in the LUAD tumor microenvironment. Interestingly, the infiltration level of certain immune cells was closely related to the expression of both two genes. As shown in Fig. 5D, though in low correlations, the B cell infiltrate was negatively associated with UHRF1 expression, while CD8 + T cells, macrophages, and neutrophil cells were positively correlated with UHRF1 expression. All six immune cells except myeloid cells were positively correlated with MECP2 expression. Besides, the patients with mutated MECP2 showed higher CD8 + T cells and neutrophil cells contents (Fig. 5E). Subsequently, GSEA was performed to identify the abnormally activated signaling pathways due to UHRF1 and MECP2 abnormal expression in LUAD. The results exhibited that some immune-related pathways like B and T cell receptor signaling pathways were closely related to both genes (Fig. 5F).

### Predictive value of DMRRS in anti-PD-1 immunotherapy

**Fig. 6:**
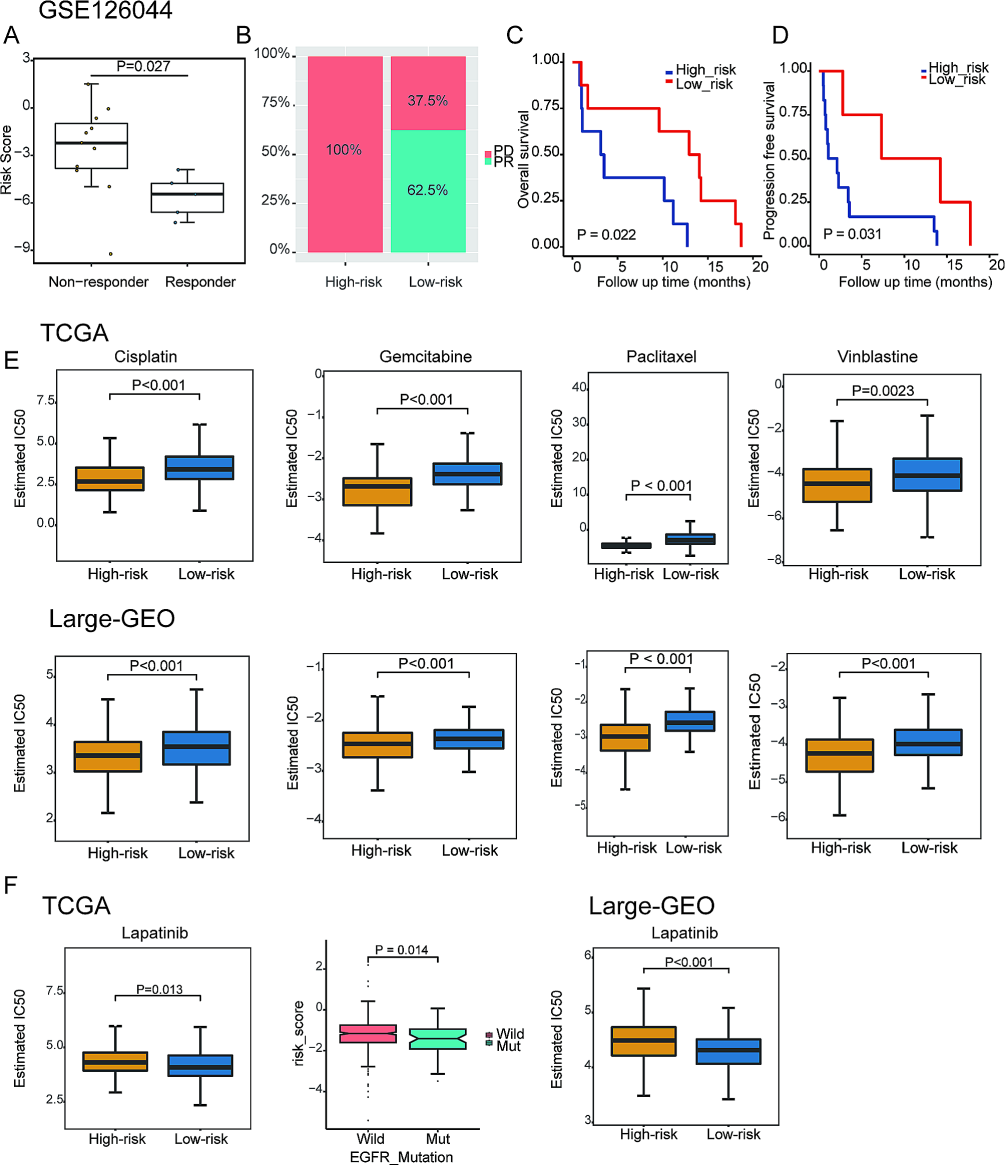
The Role of DMRRS in Anti-PD-1 Immunotherapy and Drug Sensitivity. (**A**) The risk score difference in non-responders versus responders. (**B**) The proportion of patients with response to PD-L1 blockade immunotherapy in low- or high- score groups. (**C**) OS analyses for low (8 cases) and high (8 cases) risk patients’ groups in the anti-PD-L1 immunotherapy cohort (GSE126044 cohort). (**D**) PFS analyses for low (8 cases) and high (8 cases) risk patients’ groups in the anti-PD-L1 immunotherapy cohort (GSE126044 cohort). (**E**) Distribution of the estimated IC50 of Cisplatin, Gemcitabine, Paclitaxel, and Vinblastine between high- and low-score groups in the TCGA and the Large-GEO cohort. (**F**) Distribution of the estimated IC50 of Lapatinib between high- and low-score groups in the TCGA and the Large-GEO cohort. And distribution of the risk scores in different EGFR mutation statuses in the TCGA cohort. PD, progressive disease. PR: partial response. OS: overall survival. PFS: progression-free survival

Based on the GSE126044 immunotherapy cohort, consisting of 16 NSCLC patients with the intervention of anti-PD-1 antibody Nivolumab, we evaluated the ability of the DMRRS in predicting anti-PD-1 treatment response. The risk score of responders was significantly lower than non-responders, and the proportion of patients who responded to anti-PD-1 treatment in the low-risk group was 62.5% versus 0% in the high-risk group (Fig. 6A and B). Moreover, patients with lower risk scores exhibited a markedly prolonged survival, including OS and PFS (Fig. 6C and D).

### Chemotherapy and targeted therapy sensitivity between DMRRS-defined subgroups

For LUAD treatment, chemotherapy and targeted therapy play indispensable roles and are widely used nowadays. It’s thus crucial to identify subgroup patients who could be more sensitive to some specific drugs. Therefore, we estimated the therapeutic response to several commonly used drugs in high- and low-risk group patients. The low-risk group was more sensitive to Lapatinib, while the high-risk group was more sensitive to Cisplatin, Paclitaxel, Vinblastine, and Gemcitabine in both TCGA and Large-GEO cohorts (Fig. 6E and F). As lapatinib is not routinely used in lung cancer patients, we further compared the EGFR mutation in different risk groups. Consistent with the therapeutic response, the EGFR-Mut patients had significantly lower risk scores than the EGFR-Wild patients (Fig. 6F). Altogether, the DMRRS could help us determine the personalized treatment for LUAD patients.

### Validation of predictive potential of DMRRS in prognosis and immunotherapy response

**Fig. 7:**
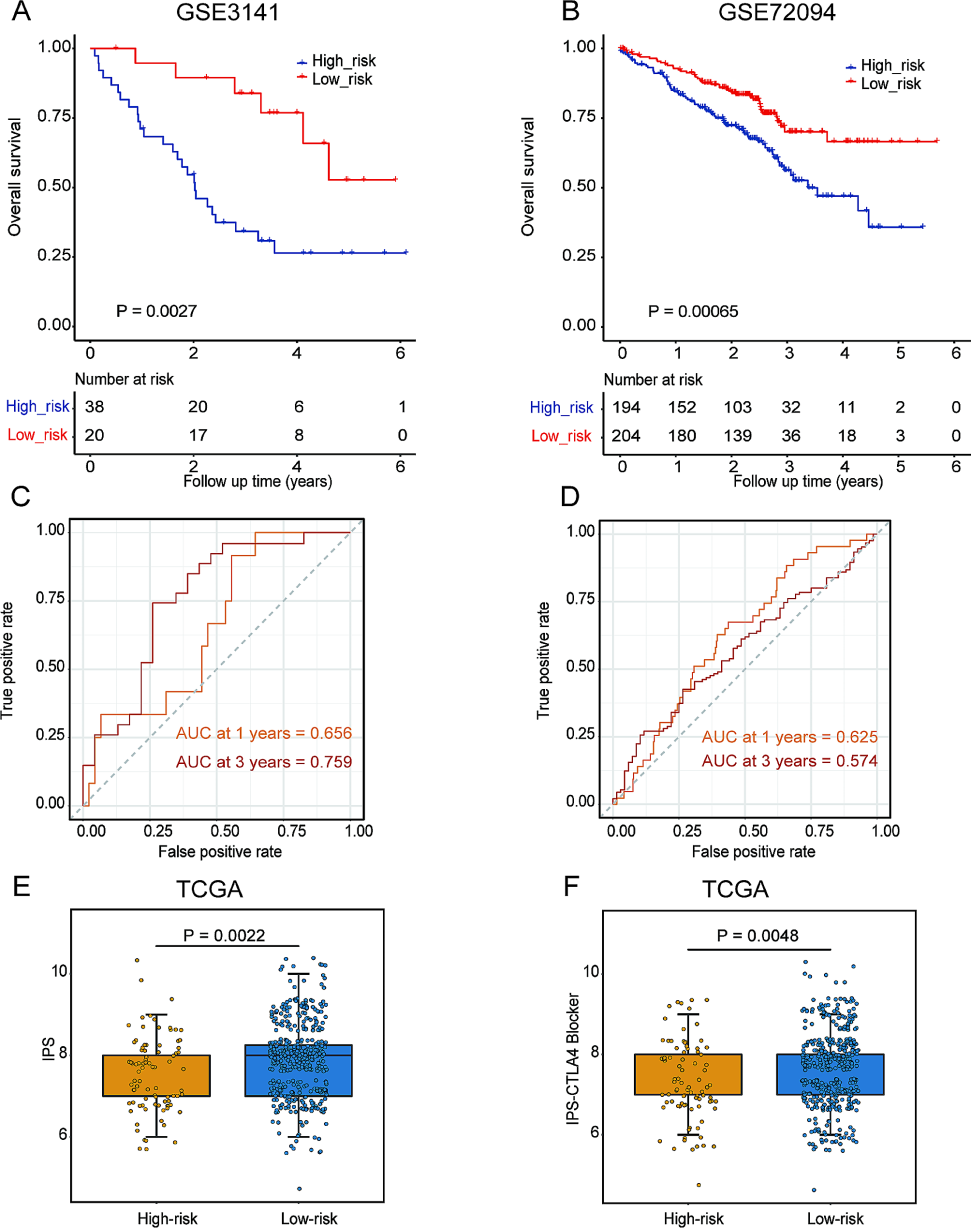
Validation of Prognosis and Predictive Potential in Immunotherapeutic Benefits of DMRRS. (**A-B**) Kaplan-Meier curves of OS in high- and low-risk groups in the GSE3141 and GSE72094 datasets. (**C-D**) ROC curve analyses in the GSE3141 and GSE72094 datasets. (**E-F**) The distribution of IPS in the high-risk and low-risk groups in the TCGA dataset

As independent validation sets, GSE3141 and GSE72094 cohorts were utilized to test the prognostic value of DMRRS further. Consistent with large-GEO and TCGA results, the low-risk groups showed longer OS than patients in high-risk (Fig. 7A and B). ROC curve exhibited that the 1- and 3- year AUC values of GSE3141 were 0.656 and 0.759, with 0.625 and 0.574 for 1- and 3- year respectively in GSE72094 (Figs. 7C and D).

Since no other immunotherapy cohort of lung cancer patients was available, we tried to validate the predictive potential of DMRRS of ICIs by exploring the correlation between DMRRS and IPS score, one recognized immunotherapy predictor. As depicted in Fig. 7E and F, low-risk patients had significantly increased IPS score and higher response rate in the anti-CTLA4 treatment.

### Validation of DMRRS-related gene expression in LUAD tissues

**Fig. 8:**
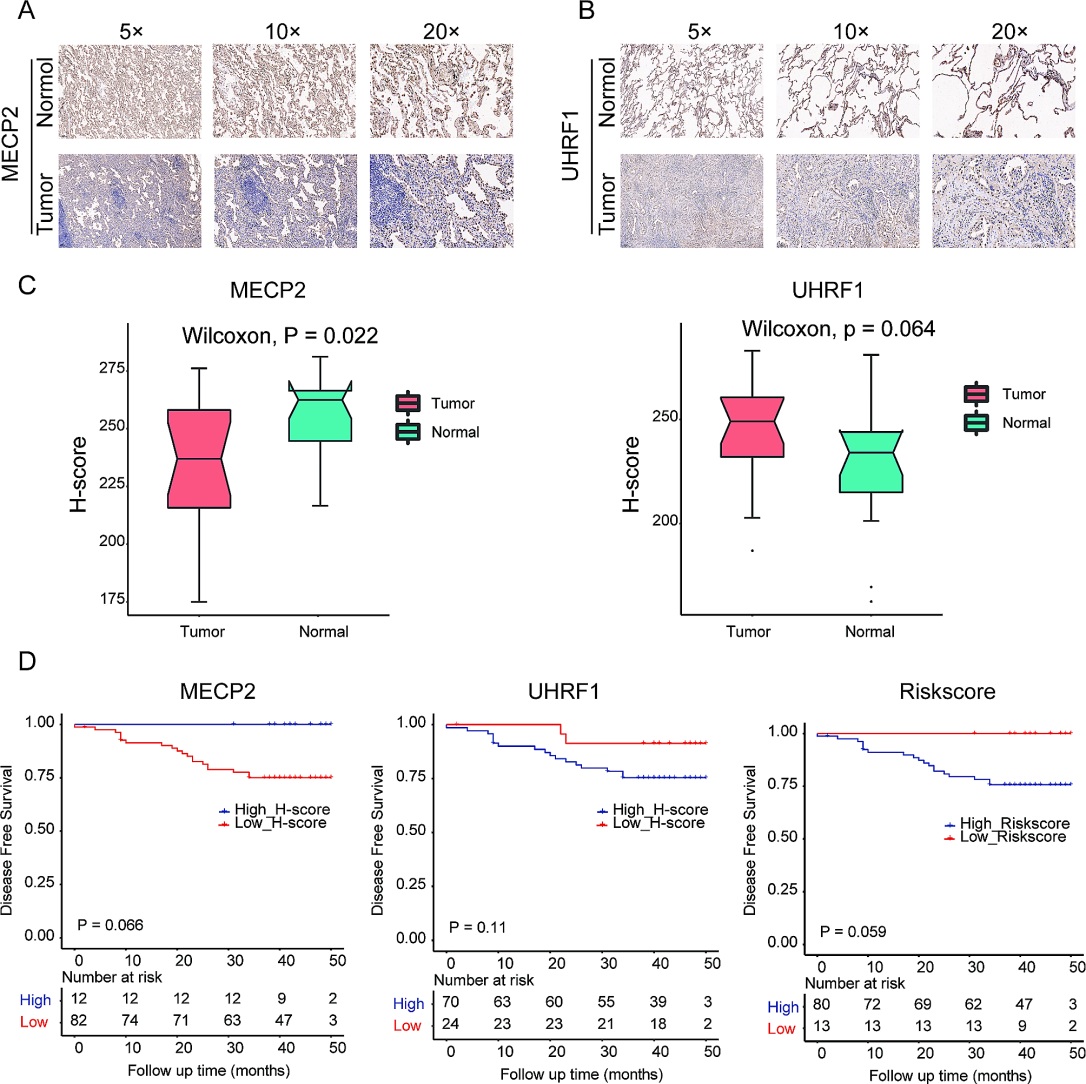
IHC validation of DMRRS Gene Expressions and survival analysis in LUAD Tissues. (**A-B**) Quantification of protein expression of MECP2 (**A**) and UHRF1 (**B**). (**C**) Comparison of protein expression of MECP2 and UHRF1. (**D**) Kaplan-Meier curves of DFS in different MECP2 and UHRF1 protein expression groups and for patients with different risk scores in our in-house dataset. DFS, disease-free survival

Both UHRF1 and MECP2 genes were differentially expressed in normal and tumor lung samples, as indicated in Fig. 2G. We quantified those two genes by IHC in 18 LUAD tumors and adjacent normal tissues. The IHC staining revealed significantly lower expression of MECP2 in tumors (*P* = 0.022). In contrast, the UHRF1 expression was relatively elevated in tumors with a *P* value of 0.064 due to the limited test samples (Fig. 8A-C).

For survival analysis, 94 patients with survival data were included. The results showed that patients with higher expression of MECP2 or lower expression of UHRF1 had longer disease-free time compared with the other group (Fig. 8D). The results were consistent with the survival analysis in the TCGA data (**Supplementary Fig. 2)**. Though the signature was constructed with RNA expression, we were curious about the survival value of the same signature formula using the IHC data. The group with higher risk scores exhibited worse DFS (Fig. 8D, *P* **= 0.059**). Interestingly, the signature performed better than both genes alone from the view of *P* value. The clinical characteristics of 94 patients are shown in **Supplementary Table **4. Most patients were at an early stage with fairly good clinical outcomes, which may contribute to the non-significant *P* value in our in-house data survival analysis.

## Discussion

DNA methylation has been widely revealed as a promising target for the development of robust prognostic and antitumor immunity biomarkers [17, 18]. However, since most studies have focused on the role of DNA methylation location, further research focusing on DNA methylation regulators is warranted to demonstrate the potential regulatory mechanism of DNA methylation.

This study comprehensively analyzed the prognostic effects, tumor microenvironment association, chemotherapy and targeted therapy drug sensitivity, and immune response of DNA methylation regulators. Most regulators were significantly abnormally expressed in tumor tissue than in the adjacent normal tissues. Then we nominated the DMRRS for reliable use as an independent prognostic factor. Concurrently, the signature was also closely related to immune scores and immune cell infiltration levels. In the immune cohort GSE126044, the DMRRS showed an impressive success in selecting responders who could benefit from ICIs. More critically, the use of the DMRRS in those settings, including predicting prognosis and immune response, had been carefully vetted and validated.

The DMRRS consists of UHRF1 and MECP2. Ubiquitin-like with PHD and ring finger domains 1 (UHRF1) is a chromatin modifier, which participates in DNA methylation and can contribute to cancer progression in many tumors, including lung cancer [9, 12, 26]. Researchers also found that by affecting the cell cycle and inducing cell apoptosis, UHRF1 could contribute to the poor prognosis in LUAD recently [36]. Methyl-CpG Binding Protein 2 (MECP2) is a member of the methyl-CpG-binding domain (MBD) family and plays a vital role in chromatin organization [15]. Accumulating studies have proved the potential roles of MECP2 in tumor progression [22, 37]. Thus, it’s reasonable to speculate that the DMRRS influences the prognosis of LUAD patients. In our study, the DMRRS did show excellent prognostic value in the Large-GEO training dataset and three validation cohorts, including TCGA, GSE3141, and GSE72094.

Growing evidence has shown that the aberrant expression of DNA methylation regulators could trigger downstream metabolism disorder and is involved in antitumor immunity regulation. For example, TET2 acted as a tumor suppressor in solid tumors and could predict patient response and the efficacy of anti-PD-1/PD-L1 therapy [39]. Based on 21 methylase-related regulators, two DNA methylation modification patterns were identified and correlated with tumor immune-infiltrating microenvironment well in lung cancer [42]. However, a robust DNA methylation regulators-related signature in immunotherapy response remained unexplored. Based on 20 DNA methylation regulators, we constructed the DMRRS that was closely related to CD8 + T cells and could effectively predict the immune response in LUAD. Since the higher IPS scores, the better immunotherapy response, we validate that the low-risk patients had higher IPS scores.

The immune checkpoint blockade therapy has revolutionized the treatment of lung cancer. Considering the good predicting potential of DMRRS in LUAD immune response, we were interested in unraveling why DMRRS can predict the immune response in LUAD. Using the TIMER databases, we revealed that UHRF1 and MECP2 were closely associated with high immune cell infiltration levels in the tumor microenvironment. Also, we performed the single-cell analysis using the TISCH database (**Supplementary Fig. **3). The GSE131907 datasets were utilized, including 58 LUAD tissues, and divided into 12 types of cells. In the database, UHRF1 had the highest infiltration level in plasma though the expression was low, and MECP2 had the highest infiltration degree in CD8 + T cells. Besides, UHRF1 was the top gene in the dendritic and plasma cells of the GSE131907 cohort. Consistent with our results, UHRF1 regulation of the Akt-mTOR pathway was essential for invariant natural killer T (iNKT) cell survival [4]. In systemic lupus erythematosus (SLE), downregulation of UHRF1 led to T follicular helper (Tfh) cell differentiation both in vitro and in vivo [21]. MECP2 was explicitly bound to the foxp3 locus, thus promoting Foxp3 expression and Tregs’ resilience [20]. These findings implied that DMRRS might shape the tumor microenvironment for immunotherapy treatment by recruiting immune cells. In fact, targeting UHRF1 in combinational immunotherapy of lung cancer has already been undergone.

In view of the clinical significance, we constructed a tool, namely DMRRS, with an excellent ability to identify LUAD patients who may live longer and benefit from immunotherapy. The predictive value was validated. We also found that the DMRRS may regulate the tumor immunity of LUAD through interacting with the tumor microenvironment immune cells.

Undeniably, there are several limitations in this study. First, there were relatively few stage IV LUAD patients in the TCGA database, which may lead to biases in subsequent studies on DNA methylation regulators and the TNM stage. Second, the samples for IHC staining weren’t enough due to a lack of budget and time. Finally, the regulatory mechanism of DNA methylation regulators in LUAD progression and tumor microenvironment was warranted to be further investigated.

## Conclusions

In conclusion, the DMRRS tool or DMRRS-related genes can be a robust biomarker for clinical outcomes and immunotherapy response.

### Electronic supplementary material

Below is the link to the electronic supplementary material.

**Supplementary Table 1**. The detailed clinical information of the TCGA-LUAD, GSE19188, GSE30219, GSE31210, GSE3141, GSE37745, GSE50081, and GSE72094 cohorts.

**Supplementary Table 2**. Univariate Cox Analysis Results of 20 DNA Methylation Regulators in TCGA.

**Supplementary Table 3**. Univariate Cox Analysis Results of 20 DNA Methylation Regulators in GEO.

**Supplementary Table 4**. Patient characteristics of our in-house data.

**Supplementary Fig. 1**. ROC curve in the GEO cohorts and survival analysis in the TCGA cohorts regarding different clinical features. (A) ROC curve of 1-, 3- and 5-years. (B-D) OS analysis between high- and low-risk subgroups in regard of different clinical features, including age, gender, and stages. (E) Correlation between high-risk groups and immune cells. ROC: Receiver operating characteristic. DFS: Disease-free survival.

**Supplementary Fig. 2**. Survival analysis of different expression groups of MECP2 and UHRF1 in the TCGA cohort. (A) OS analysis between high- and low-expression subgroups of MECP2 and (B) UHRF1 in TCGA-LUAD cohort. OS: Overall survival.

**Supplementary Fig. 3**. The single-cell analysis using the TISCH database. (A) The cell types and their distribution in the GSE131907 of the TISCH database. (B) The pie chart showed different cell types and their distribution in the GSE131907 cohort. (C and D) The expression of UHRF1 and MECP2 in different immune cells in the GSE131907 dataset. (E and F) The distribution of UHRF1 and MECP2 in different cell types in the GSE131907. TME: tumor microenvironment; TISCH: Tumor Immune Single Cell Hub.

## Data Availability

Data used in this study from the TCGA and GEO databases can be accessed without restriction. In-house immunohistochemistry (IHC) images can be accessible through email to the corresponding author Songhua Cai on reasonable request.
