# Supplementary figures and images for: Comprehensive analysis reveals the prognostic and immunogenic characteristics of DNA methylation regulators in lung adenocarcinoma
Source: Respir Res. 2024 Feb 5;25:74. doi: 10.1186/s12931-024-02695-4 (PMC10845581; doi:10.1186/s12931-024-02695-4)

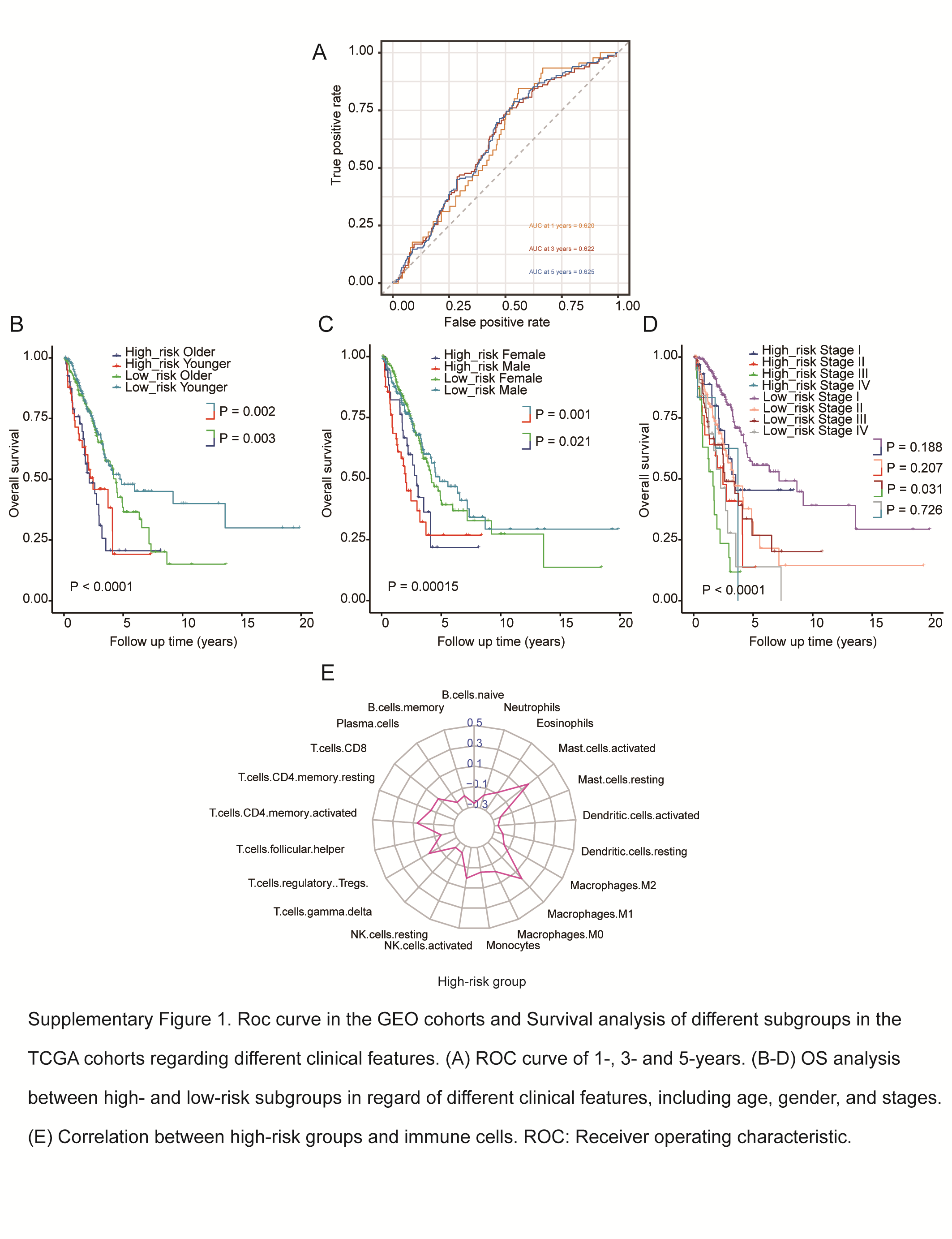

Supplement: Supplementary file 5 — Supplementary Fig. 1. ROC curve in the GEO cohorts and survival analysis in the TCGA cohorts regarding different clinical features. (A) ROC curve of 1-, 3- and 5-years. (B-D) OS analysis between high- and low-risk subgroups in regard of different clinical features, including age, gender, and stages. (E) Correlation between high-risk groups and immune cells. ROC: Receiver operating characteristic. DFS: Disease-free survival. [file 12931_2024_2695_MOESM5_ESM.png]

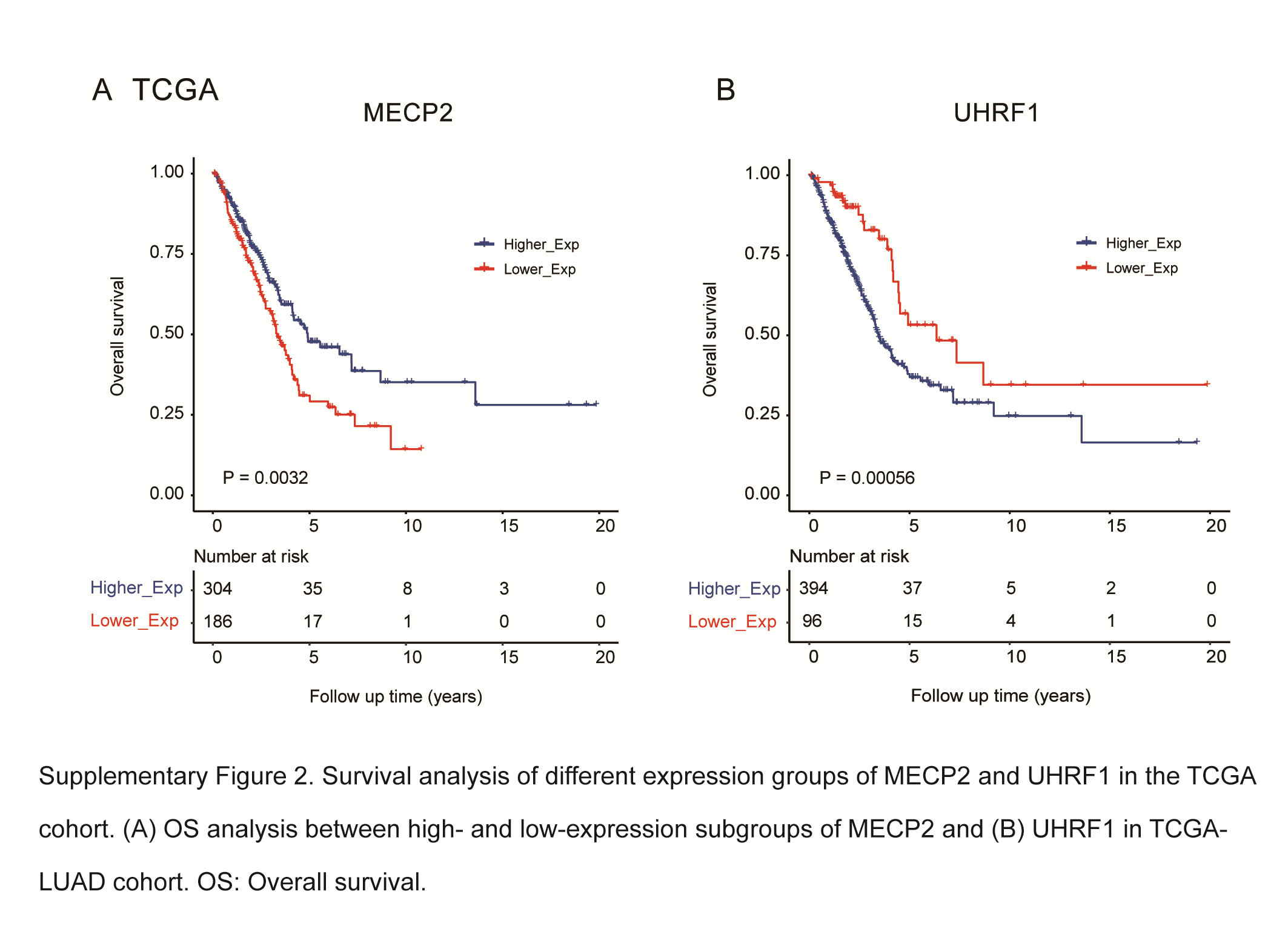

Supplement: Supplementary file 6 — Supplementary Fig. 2. Survival analysis of different expression groups of MECP2 and UHRF1 in the TCGA cohort. (A) OS analysis between high- and low-expression subgroups of MECP2 and (B) UHRF1 in TCGA-LUAD cohort. OS: Overall survival. [file 12931_2024_2695_MOESM6_ESM.png]

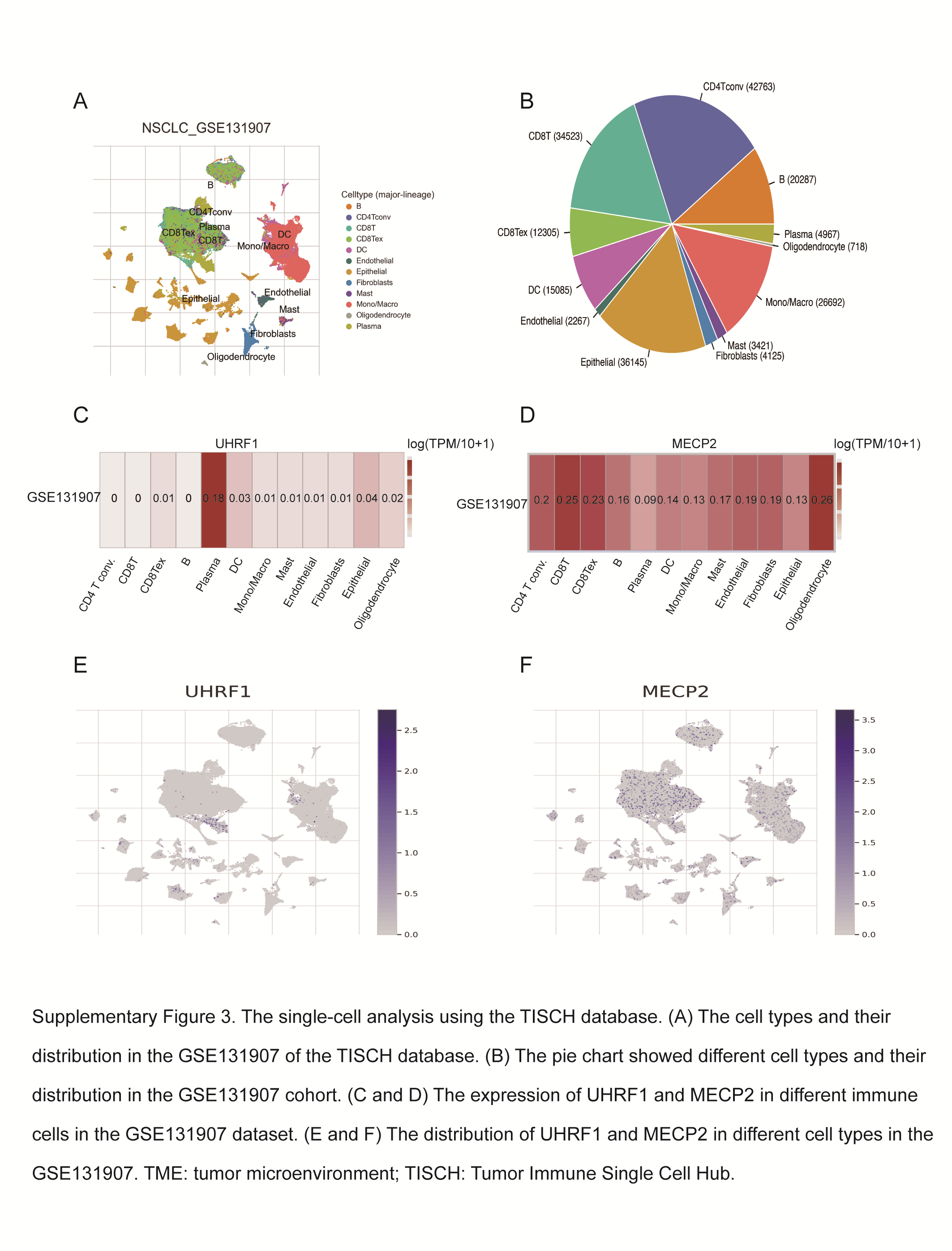

Supplement: Supplementary file 7 — Supplementary Fig. 3. The single-cell analysis using the TISCH database. (A) The cell types and their distribution in the GSE131907 of the TISCH database. (B) The pie chart showed different cell types and their distribution in the GSE131907 cohort. (C and D) The expression of UHRF1 and MECP2 in different immune cells in the GSE131907 dataset. (E and F) The distribution of UHRF1 and MECP2 in different cell types in the GSE131907. TME: tumor microenvironment; TISCH: Tumor Immune Single Cell Hub. [file 12931_2024_2695_MOESM7_ESM.png]
